# Discrimination in measures of knowledge monitoring accuracy

**DOI:** 10.5709/acp-0161-y

**Published:** 2014-09-30

**Authors:** Christopher A. Was

**Affiliations:** Educational Psychology Laboratory, Kent State University, USA

**Keywords:** knowledge monitoring, metacognition, measures of knowledge monitoring

## Abstract

Knowledge monitoring predicts academic outcomes in many contexts. However, measures of knowledge monitoring accuracy are often incomplete. In the current study, a measure of students’ ability to discriminate known from unknown information as a component of knowledge monitoring was considered. Undergraduate students’ knowledge monitoring accuracy was assessed and used to predict final exam scores in a specific course. It was found that gamma, a measure commonly used as the measure of knowledge monitoring accuracy, accounted for a small, but significant amount of variance in academic performance whereas the discrimination and bias indexes combined to account for a greater amount of variance in academic performance.

## Introduction

The current investigation was designed to test the efficacy of measures of knowledge monitoring accuracy in the context of a simple assessment of knowledge monitoring. Performance on many versions of this knowledge monitoring assessment has been linked to academic outcomes (e.g., Hartwig, Was, Isaacson, & Dunlosky, 2012). Gamma (γ) has been the measure of choice to assess individual differences in knowledge monitoring accuracy. This investigation examines the relationship between other measures derived from signal detection theory (*d*’ and lamda [λ]), gamma, and academic performance.

*Metacognition* is often described as *thinking about one’s thinking*. Although in layman’s terms this is a reasonable definition, a more precise definition of metacognition is *knowledge and control of one’s cognitive processes* (Flavell, 1979). There is a great deal of research in cognitive psychology and educational psychology that demonstrates a strong link between metacognition and learning (Dunlosky & Metcalfe, 2009; Hacker, Dunlosky, & Graesser, 2009).

Models of metacognition typically include monitoring as an essential component of learning (Dunlosky & Metcalfe, 2009; Nelson & Narens, 1990; Tobias & Everson, 2009). For example, Nelson and Narens described a model of metacognition composed of two levels: the meta-level and the object level. The object level represents ongoing cognitive processes involved in completing a task (e.g., learning and attention). The meta-level contains a model of the person’s understanding of the task at hand and the cognitive process involved in attempting to complete the task. The processes involved in metacognition are the interactions that occur between the meta-level and the object-level. These two processes are monitoring and control. Monitoring represents the meta-level’s knowledge and appraisal of the object level. Put differently, the object-level informs the meta-level of the ongoing cognitive activities so the meta-level can update the model. Control represents the meta-level updating the activities in the object-level. As specified by Nelson and Narens, control of the object level does not provide information about the ongoing activities, and therefore monitoring is a necessary and foundational aspect of metacognition.

Tobias and Everson (2009) presented a hierarchical model of metacognition and suggested that knowledge monitoring is the foundation of metacognition. Only with accurate knowledge monitoring can one successfully employ more complex metacognitive process such as planning, evaluation, and selecting learning strategies.

In an attempt to empirically measure students’ ability to accurately monitor their knowledge Tobias, Hartman, Everson, and Gourgey (1991; see also Tobias & Everson, 2002) devised a simple assessment to measure students’ knowledge monitoring ability. This simple knowledge monitoring assessment requires participants to make judgments about their knowledge. For example, participants may be asked whether they know the meaning of each word in a list of words. Participants simply respond to each word by stating, “yes, I know the meaning of the word,” or “no, I do not know the meaning of the word.” These judgments are often referred to as *confidence judgments*. After completing this series of confidence judgments participants’ knowledge of the items on the word list is then assessed. In this example, they would be administered a multiple-choice test of the definitions of the words on the list. Accuracy on the multiple-choice test is a performance outcome. Performance (or the precision with which students identify correctly answered multiple choice items as “known” and incorrectly answered items as “unknown”) on the knowledge monitoring assessment (KMA) and similar assessments show clear connections between knowledge monitoring accuracy and academic achievement (see Tobias & Everson, 2009, for a review).

Indeed, several studies using undergraduate students as participants have demonstrated that general knowledge monitoring ability measured at the beginning of a semester predicts achievement throughout or at the end of the semester. For example, Hartwig et al. (2012) found that students’ general knowledge monitoring accuracy at the beginning of a semester-long course correlated with their final exam scores in that same course. Isaacson and Was (2010) also found that a simple knowledge monitoring assessment administered at the beginning of the semester accounted for variance in final exams scores. Although Isaacson and Was’s goal was to demonstrate an increase in knowledge monitoring accuracy after training in a course, their results indicated that knowledge monitoring accuracy at the beginning of the semester accounted for as much variance in final exam performance as knowledge monitoring accuracy at the end of the semester. Was, Beziat, and Isaacson (2013) also replicated these results in a study examining improvements in monitoring accuracy following training to increase knowledge monitoring. Clearly this line of research has demonstrated that individual differences in knowledge monitoring accuracy, as measured by a simple and independent assessment, are related to performance within a course.

Tobias and Everson (2009) reviewed a number of studies that indicate that differences in knowledge monitoring accuracy also predict differences in more long-term academic measures, such as grade point average and SAT® (formerly known as the Scholastic Aptitude Test) scores. Although convincing evidence exists that knowledge monitoring accuracy is an important component of metacognition, the question remains: To what extent are individual differences in response patterns related to knowledge monitoring accuracy?

To understand how individual differences might influence accuracy of responses on a knowledge monitoring assessment it is necessary to understand the responses generated and how they are analyzed and interpreted. For the purposes of the KMA, the proportion of items responded to correctly (i.e., items answered correctly) is not directly relevant. Rather than relying on the proportion of correct responses, the results of the KMA are typically interpreted based on the proportion of items correctly identified as known or unknown. For example, if an item is identified as unknown during the initial phase and is responded to incorrectly during the testing phase this would be identified as accurate knowledge monitoring.

The results of the KMA are most often presented in the form of a 2 × 2 contingency table similar to the one shown in Table 1. Cells *a* and *d* represent correctly identified items based on the responses during the initial phase and subsequent results during the test phase, and hence represent accurate knowledge monitoring. Conversely, cells *b* and *c* represent misidentified items and thus ineffective knowledge monitoring.

**Table 1.** Accuracy Assessments Generated by the Knowledge Monitoring Assessment

	Response accuracy	
Response	Correct response	Incorrect response
	*a*	*b*
Know	Hits	False alarms
	*c*	*d*
Don’t know	Misses	Correct rejections

Put differently, the procedure generates the following four scores with participants assessing the item: (a) known and correctly responded to the item on the vocabulary test (hits); (b) known but responded to incorrectly on the test (false alarms); (c) unknown but the correct response was given on the test (misses); and (d) unknown and responded to incorrectly on the test (correct rejections).

There are a number of ways to analyze the results of knowledge monitoring data, including those that generate a 2 × 2 contingency table as above. It is important to distinguish between *absolute accuracy* and *relative accuracy*. *Absolute accuracy*, also known as *calibration*, represents how closely a judgment of performance corresponds to actual performance. Put differently, absolute accuracy measures whether one can predict test performance (Dunlosky & Metcalfe, 2009). An individual’s calibration would be perfect if she or he predicted to answer 75% of the items on a test correctly and they did answer 75% of the items (no more, no less) correctly.

*Relative accuracy*, also known as *resolution*, indicates whether an individual can differentiate between items that are known versus unknown. Put differently, resolution indicates whether metacognitive judgments of individual items predict performance relative to one another.

Nelson (1984), after a thorough review of measures of feeling-of-knowing accuracy, proposed that γ (Goodman & Kruskal, 1954) is the best measure for use with feeling-of-knowing data that align with 2 × 2 tables, but also for *R* × *C* in which *R* > 2 and *C* > 2. The studies previously described in this manuscript, as well as many other previous studies, have relied on a γ coefficient to assess the degree of accuracy on the knowledge monitoring assessment (e.g., Hartwig et al, 2012). γ in these circumstances is a measure of relative accuracy. As previously stated, measures of relative accuracy provide information about the discrimination of a set of confidence judgments in relation to a set of performance outcomes (Schraw, 2009b). Although provides a measure of monitoring accuracy, it does not account for variation in participant responses due to issues, such as response bias, poor discrimination, or lack of sensitivity. Put differently, γ whereas produces an index of accurate monitoring, it does not account for an individual’s general response tendencies (e.g., over- or underconfidence). γ is used widely in the metacognition literature, including laboratory studies investigating the accuracy of judgments of learning (JOL’s; cf. Dunlosky & Metcalfe, 2009) and in classroom studies (e.g., Hartwig et al., 2012). However, may not account for the individual differences found in knowledge monitoring accuracy that affect accuracy in both laboratory settings, and more importantly ecologically valid studies, such as those that take place in the classroom (Masson & Rotello, 2009).

For example, in the previously referenced studies conducted by Was and colleagues (e.g., Hartwig et al., 2012; Was et al., 2013) γ as the measure of knowledge monitoring accuracy did account for a significant amount of variance in classroom achievement as measured by a final examination. However, the amount of variance accounted for was relatively small (*r* values between .26 and .42). A multitude of variables play a role in classroom performance. When one considers the confounding variables in K-12 and college classrooms, the ability to account for 7% of the variance in final exam scores is quite impressive. However, the question remains: If knowledge monitoring accuracy is the foundation of metacognition, is there a measure of knowledge monitoring accuracy that might capture individual differences that impact knowledge monitoring accuracy and therefore account for more variance in academic performance?

Schraw (2009a) described five different outcome measures available when one’s goal is to measure metacognitive monitoring:

1. *Absolute accuracy index* is the difference between a confidence judgment and performance and is a measure of judgment precision.

2. *Relative accuracy* (correlation coefficient) is the relationship between a set of confidence judgments and performance scores and is a measure of the correspondence between confidence judgments and performance. Put differently, relative accuracy measures the precision of confidence judgments.

3. *Bias index* captures the degree of over- or underconfidence in judgments and includes the direction of judgment error.

4. *Scatter index* is a measure of the differences in variability for confidence judgments for correct and incorrect items.

5. *Discrimination* captures the participants’ ability to discriminate between confidence for correct and incorrect items. Put differently, discrimination captures the difference in accuracy for confidence of correct items versus confidence for incorrect items.

The bias index, scatter index, and discrimination are each possible candidates for capturing individual differences in responses. However, there are reasons that each of these is not appropriate for use in the current study (cf. Schraw, 2009a). Many of these measures are not appropriate for measuring a participant’s ability to discriminate between known and unknown items. For example, the bias index captures the degree to which the participant is more likely to answer “known” when the item is unknown (overconfidence) than to answer “unknown” to known items (underconfidence).

One candidate is the discriminability index or *d*’. *d*’ is a theoretical value used in signal detection theory that measures how readily a signal can be detected (Wickens, 2002). In signal detection theory, *d*’ measures the separation between the signal and the noise (no signal) distributions using the noise distribution’s standard deviation as the metric. Both distributions are Gaussian in nature and assumed to be of equal variance. One goal of the current study was to determine if using *d*’ to analyze data from a 2 × 2 contingency table based on a simple KMA would account for more variance in an achievement measure than using *d*’, and thus provide insight into potential individual differences that may affect knowledge monitoring and metacognition and in-turn, classroom performance.

A second goal was to determine if *response bias*, the degree to which an individual is over- or underconfident in their judgments, impacts the efficacy of *d*’ to account for variance in performance. In signal detection theory, λ is a measure of observer’s response criterion. Put differently, λ is a measure of an individual’s propensity to say “yes” or “no.” According to Wickens (2002), λ is the most direct way to describe the placement of the observer’s criterion. However, for the researcher to interpret the criterion the relationship between λ and *d*’ must be taken into account. For example, if **d*’* = 0.03 and λ = .05, this represents a bias toward *no* or *unknown* responses, but if *d*’ = 2.0 and λ = .05, this represents a bias toward *yes* or *known* responses.

Wickens (2002) and others (e.g., Macmillan & Creelman, 2005) have argued that a better measure of bias is λ_center_ (Macmillan & Creelman denote λ_center_ as *c*). λ and λ_center_ both refer to the same criterion but they differ in the origin from which the criterion is measured (Wickens, 2002). In the current study, both measures of bias were calculated as response bias may impact the relationship between *d*’ as a measure of knowledge monitoring accuracy and performance on in-class exams.

## Method

### Participants

Three hundred and sixty one undergraduates enrolled in an educational psychology course at a Midwestern University participated for course credit. Females represented 74% and males 26% of the sample. Participants in the study were freshmen and sophomores enrolled in the course as a requirement into the teacher education program at the university.

### Materials

#### Knowledge Monitoring Assessment (KMA)

The KMA used for this study was adopted from Tobias and Everson (1996; for a review, see Tobias & Everson, 2009). The KMA designed for the current study required participants to state whether they knew or did not know the meaning of 50 English words, and then respond to a multiple-choice vocabulary test of the same words (see Appendix A for stimuli).

#### Final Exam

The final exam was a 100-item, cumulative, multiple-choice exam in the educational psychology course in which the participants were registered.

### Design and procedure

Participants completed the KMA during the first two weeks of the semester. Participants logged into the online course delivery system used by the university and initiated the KMA. Participants were informed that performance on the KMA was not related to their course credit and that they should complete the KMA without using any outside resources (e.g., the textbook, a dictionary, online resources, etc.). Once participants began the KMA it was to be completed in a single session. Participants were presented with 50 vocabulary words, one at a time. Thirty-three of the words represented vocabulary items derived from the text used for the educational psychology course (content specific) and 17 represented general vocabulary items.^1^ When each of the 50 items was presented, participants were required to indicate whether they knew or did not know the meaning of the word. Participants had unlimited time to respond. After all 50 items were presented participants were presented with a multiple-choice test of the vocabulary items. Again, each vocabulary item was presented one at a time along with five possible synonyms (four distractors and one true synonym). Participants responded by indicating which of the five alternatives they believed to be the synonym. Participants had unlimited time to respond.

The final exam was administered on the last day of semester. The exam consisted on 100 multiple-choice items and was a cumulative assessment of students’ knowledge of course content.

Recall that using terminology common to signal detection theory, the procedure generates the following four scores with students assessing the words: (a) known and correctly responded to the item on the vocabulary test (hits); (b) known but responded to incorrectly on the test (false alarms); (c) unknown but the correct response was given on the test (misses); and (d) unknown and responded to incorrectly on the test (correct rejections).

To test whether *d*’ is a better predictor of academic outcomes than γ, the KMA was scored using both the γ coefficient and *d*’. The equation for calculating γ is presented in Formula 1, and for calculating *d*’ in Formula 2.

(1) $$\gamma =\frac{\mathrm{ad}-\mathrm{bc}}{\mathrm{ad}+\mathrm{bc}}$$

(2) d'=z(H)−z(F)

Equation 1 refers to the cells found in the 2 × 2 contingency table (Table 11). In Equation 2, *H* = *P*(“Known”/Correct; [*a*/(*a* + *c*)]) or the proportion correct responses identified as known, and *F* = *P*(“Known”/Incorrect; [*b*/(*b* + *d*)]) or the proportion of incorrect responses identified as known. *z* represents the inverse of the cumulative Gaussian distribution (Wickens, 2002). Table 2 presents the 2 × 2 contingency table with means and standard deviations for each of the above scores. Two participants had no data in the *misses* cell of the table. In Formula 2, participants with no data in the *misses* (*c*) cell will have a hit rate of 1. Because *d*’ is unidentified in cases in which *H* or *F* are 1 or 0, the data from these two participants was not included in the analyses to follow. Although Macmillan and Creelman (2005) suggested ways to deal with empty cells, I chose to exclude the data of these two participants as the loss of two participants translates to a loss of less than 0.01% of the data.

**Table 2.** Means and Standard Deviations of Accuracy Assessments of Knowledge Monitoring Assessment

	Response accuracy	
Response	Correct response	Incorrect response
	17,91 (7,72)	7,83 (4,40)
Know	Hits	False alarms
	8,88 (4,23)	15,15 (6,47)
Don’t know	Misses	Correct rejections

*Note*. Standard deviations in parentheses.

To understand the influence of response bias, λ the and the λ_center_ bias index (cf. Wickens, 2002) were calculated using Equation 3a and Equation 3b, where, as above, *F* = *P*(“Known”/Incorrect; [*b*/(*b* + *d*)]).

(3a) λ=−z(F)

(3b) $$\lambda_{\mathrm{center}}=\lambda -\frac{1}{2}d'$$

## Results

Table 3 displays the ranges, means, and standard deviations of final exam performance, γ, *d*’, the λ, λ_center_, and proportion of items correct for the general and content related vocabularies items in the KMA. As is evident from the descriptive statistics the final exam was challenging (*M* = .72, *SD* = .12) as were the vocabulary items presented as part of the KMA (means and standard deviations for general vocabulary and content-specific vocabulary proportion correct were *M* = .49, *SD* =.18, and *M* = .63, *SD* = .16, respectively).

**Table 3.** Descriptive Statistics of Key Variables

Variable	Range	Mean	*SD*
Final exam	27,00–95,00	72,32	12,14
γ	-.57–1,00	.58	.25
*d*’	-2,49–2,63	.20	.85
λ_center_	-.1,55–1,23	.01	.43
General vocabulary	.06–1,00	.49	.18
Content-specific vocabulary	.21–1,03	.63	.16

Because *d*’ and λ are statistically independent, as are *d*’ and λ_center_, when the two distributions are normal, it is also important to note that the distributions of *d*’ and λ were both normal. Skewness and kurtosis for *d*’, λ, λ_center_ were all within acceptable ranges (see Table 4).

**Table 4.** Descriptive Distribution Statistics for λ , λ_center_, and *d*’

Variable	Skewness	Kurtosis	Kolmogorov-Smirnov *Z*
*d*’	-.09 (.13)	.30 (.26)	.52
λ	-.15 (.13)	.15 (.26)	.81
λ_center_	-.20 (.13)	.44 (.26)	.20

*Note*. Standard errors in parentheses.

Table 5 presents the zero order correlations among the variables of interest. The correlation between λ and λ_center_ was large, as was to be expected (*r* = .74, *p* < .01). The correlation between γ and *d*’, and γ and λ the was *r* = .19 (*p* < .001). The correlations between *d*’ and λ, and *d*’ and λ_center_ were *r* = .11 (*p* = .04) and *r* = .09 (*p* = .09). The correlation between and the final exam in the course was *r* = .26(*p* < .001), the correlation between *d*’ and final exam was *r* = .34 (*p* < .001), and the λ and the final exam was *r* = .08 (*p* = .14). and *d*’, as measures of monitoring accuracy, were related to final exam scores. Whereas λ was not related to final exam performance (*r* = .07, *p* = .16), λ_center_ was (*r* = -.22, *p* < .01). Although γ and λ had a significant yet small correlation, λ did not affect the relationship between γ and final exam score. A partial correlation between γ and final exam score controlling for λ (*r* = .23, *p* < .001) revealed that the zero-order correlation between γ and the final exam score was not impacted after controlling for λ. The zero-order correlation between γ and λ_center_ was insignificant.

**Table 5.** Correlations Among Observed Variables

Variable	1	2	3	4	5	6	7	8	9	10
1. Final exam	1,00									
2. γ	.26	1.00								
3. *d*’	.34	.19	1.00							
4. λ	.08	.11	.74	1.00						
5. λ_center_	-.22	.03	.09	.74	1.00					
6. Vocabulary	.40	.20	.93	.46	-.25	1.00				
7. Hits	.45	.38	.65	.01	.85	-.64	1.00			
8. Misses	-.07	-.32	.54	.85	.30	-.25	.71	1.00		
9. False alarms	-.15	-44	-.72	-.87	-.54	-.24	-.56	-.55	1.00	
10. Correct rejections	-.38	.07	-.62	.03	-.82	-.85	.02	-.03	.66	1.00

*Note*. Final exam and Vocabulary are proportion of correct responses. All correlation values in table .15 or greater *p* < .01.

Another way to determine if response bias (such as overconfidence) affects the relationship between γ and exam performance is to examine the correlations between false alarms and misses, and final exam scores. As false alarms represent overconfidence and misses represent underconfidence, an individual’s tendency to have more false alarms or more misses may affect the relationship between γ and exam performance. Zero-order correlations indicate that whereas misses did not correlate to exam performance (*r* = -.07, *p* = .22),false alarms were correlated with exam performance (*r* = -.15, *p* = .005).This relationship indicates that the tendency to be overconfident regarding one’s knowledge has a negative impact on one’s performance in the classroom. Indeed, a Sobel test of mediation indicated that the relationship between γ and final exam score was mediated by false alarms, *Z*_Sobel_ = 2.73, *p* = .006.

Although the correlations between γ, *d*’, and the final exam were significant, it is important to note that performance scores on exams, such as the final exam in this study, are often predicted by vocabulary knowledge. Furthermore, performance on the KMA is heavily dependent upon vocabulary knowledge. Referring to Table 5, one can see that the correlation between γ and the proportion of correctly identified vocabulary items (vocabulary accuracy) is *r* = .20(*p* < .001). The correlation between *d*’ and the vocabulary accuracy is *r* = .93 (p < .001), indicating that in these data, *d*’ and vocabulary accuracy share a large amount of variance. However, one goal of the current manuscript is to determine if *d*’ as a measure of knowledge monitoring accuracy can account for unique variance in performance on an independent measure of classroom performance. As can be seen in Table 5, *d*’ and vocabulary accuracy both correlated with final exam performance (*r* = .34 and *r* = .40, respectively). A test of mediation by vocabulary accuracy on the relationship between *d*’ and final exam score revealed that vocabulary accuracy significantly attenuated the relationship between *d*’ and final exam score, *Z*_Sobel_ = 2.36, *p* = .02. This is not a surprising finding as there is clear evidence that general intelligence (“g”) predicts correlations between seemingly unrelated measures of vocabulary and knowledge (e.g., Frey & Detterman, 2004).

**Figure 1.**
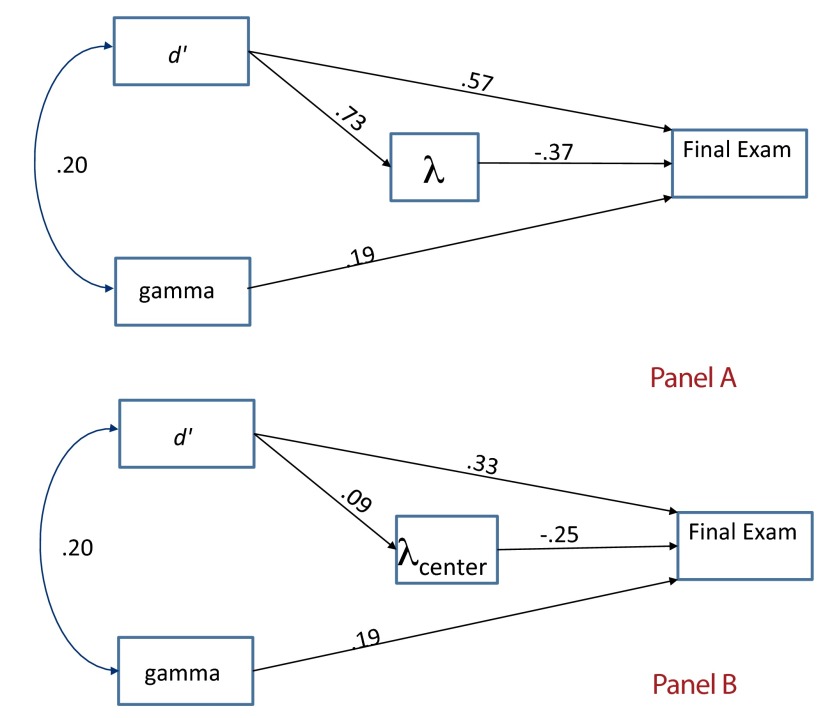
Final tested path model with standardized parameter estimates.

As noted above, the current data indicated a strong correlation between λ and *d*’. This relationship is addressed in a path model. To test the ability of γ and *d*’ as measures of knowledge monitoring to predict academic outcomes, a path model was developed and tested. A first attempt at a path model included vocabulary accuracy, but due to the large amount of shared variance between vocabulary accuracy and *d*’ the model was not a good fit to the data. Panel A of Figure 1 displays the final tested model with standardized parameter estimates. All parameters are significant and based on calculated fit indices the model is an excellent fit to the data, *x*^2^(1) = 1.29, *p* = .27, RMSEA = .025,CFI =.999.

Total standardized effects of γ and *d*’ on final exam performance were β = .31 and β = .19, respectively. Although the standardized direct effects of *d*’ were larger (β = .57), this relationship was attenuated by the negative effects of λ on final exam performance (β = -.25). There is also evidence that λ acted as a suppressor variable regarding the relationship between *d*’ and final exam score. Zero-order correlations indicated no relation between λ and final exam score and a smaller relation between *d*’ and final exam score. However, when these variables were placed into a regression model the relationships among these variables changed in a dramatic way. The model indicates that although γ accounts for unique variance in final exam performance, *d*’ and λ each account for unique variance, and reveal an interdependent relationship.

Panel B of Figure 1 displays the same model with λ replaced by λ_center_. All parameters are significant, with the exception of the path between *d*’ and λ_center_. The model is an excellent fit to the data, *x*^2^(1) = 1.07, *p* = .30, RMSEA = .014, CFI = .999. As with the model using λ, in the model using λ_center_ total standardized effects of γ and *d*’ on final exam performance were β = .31 and β = .19, respectively. In this case the standardized direct effects of *d*’ (β = .33) were again attenuated by the negative effects of λ_center_ on final exam performance (β = -.25). Again, the model indicates that although γ accounts for unique variance in final exam performance, *d*’ and λ_center_ each account for unique variance, and reveal an interdependent relationship.

## Discussion

The results of the current study clearly indicate that *d*’ accounted for unique variance in final exam scores that was not accounted for by γ, regardless of the measure of bias included in the model. Although γ has been widely used as a measure of accurate know-ledge monitoring in the past it only accounted for 3% of the variance in final exam scores in this study. *d*’, however, accounted for 9% of variance in final exam scores. For both measures it can be argued that these are important findings. After measuring students’ know-ledge monitoring accuracy in the first 2 weeks of the semester, both measures were still predictive of final exam scores 16 weeks later. When one considers all of the variables that might predict final exam performance, this amount of variance accounted for is impressive.

The relationships between *d*’, λ, and λ_center_ in the current data are an important finding in regards to the knowledge monitoring assessment. As described by Wickens (2002), in signal detection theory *d*’ is a measure of how readily a signal can be detected. In perceptual and diagnostic tasks in which signal detection is employed, *d*’ is an estimate of the signal strength and is independent of the criterion the participant adopts. To increase discriminability one simply needs to increase the signal strength. In studies attempting to measure individual differences in knowledge monitoring accuracy, such as the current study, the *signal* is each participant’s feeling of sense of correctness.^2^ A stronger signal is more easily detected. One interpretation of a *stronger signal* in this case is information that is well known or well learned. Therefore, those who can more readily recognize and detect information as known even when it is not readily available or known, will be better at knowledge monitoring.

However, response criterion, as measured by λ, and bias, as measured by λ_center_, capture the propensity to say “yes” or “no,” in this study “known” or “not known.” In the case of the KMA, λ as the criterion for “known” responses is difficult to interpret because it requires one to take detectability (*d*’) into account. However, because λ_center_ is more easily interpreted (greater values for λ_center_ represent a greater likelihood of responding “unknown”), it represents a more precise measure of bias. The negative correlation between λ_center_ and final exam scores is simply interpreted as the more students are overconfident in their knowledge, the more poorly they will perform on tests of their knowledge. It is my conclusion that this represents the overconfidence seen in many studies of knowledge monitoring and calibration in which we see the lowest performers are often overconfident in their ability to perform.

In regards to γ, false alarms as a measure of overconfidence (the tendency to respond to an item as “known” and fail to correctly identify the item) mediated the relation between γ and final exam scores. In the context of a student preparing for an exam it is clear how false alarms would impact performance. First, from a measurement perspective, false alarms will reduce the magnitude of γ. Put differently, more false alarm errors necessarily reduce the accuracy of knowledge monitoring. A student preparing for an exam, who is overconfident in his or her knowledge, is therefore likely to end the studying process prematurely. This bias toward overconfidence will in turn reduce the effect of knowledge monitoring on academic performance.

Metacognition is an important part of self-regulated learning and knowledge monitoring is the foundation of metacognition. Imagine a student preparing for an upcoming examination. If the student is able to accurately assess what she knows, she will use her time efficiently by not studying material that has been learned. However, imagine a student with poor knowledge monitoring ability. This student may inaccurately judge content to be unlearned that he has already mastered and waste time studying the material. But even more damaging to a student’s potential success is when material that is not yet mastered is judged as known, and the student stops studying prematurely. Several studies have demonstrated that these overconfident students are likely to perform poorly on examinations (e.g., Hacker, Bol, Horgan, & Rakow, 2000; Isaacson & Fujita, 2001).

One potential reason for this overconfidence is the unskilled and unaware hypothesis (Ehrlinger, Johnson, Banner, Dunning, & Kruger, 2008; Kruger & Dunning, 1999). The *double-curse*, as it is often called, occurs because not only do poor performers lack the skill to produce accurate responses (i.e., correctly answer exam questions), but they also lack the expertise to know they are not producing accurate responses (i.e., they are unable to judge the quality and accuracy of their responses). Clearly, individual differences in this ability to accurately assess knowledge are linked to academic success.

The current study was conducted as an attempt to investigate two possible measures of differences in knowledge monitoring accuracy. γ, a common measure of data that can be arranged in a 2 × 2 contingency table, has been the most often used measure of knowledge monitoring accuracy with such data. The current study supports the use of γ as it successfully accounted for individual differences in knowledge monitoring accuracy that may lead to differences in academic performance. However, the amount of variance of which γ accounted was limited.

In the current study, it was demonstrated that *d*’ was highly correlated with vocabulary knowledge. It was also found that *d*’ in conjunction with λ accounted for a larger amount of variance in final exam performance than γ.

By no means does the current investigation attempt to account for all differences in knowledge monitoring. Indeed, Schraw, Kuch, and Gutierrez (2012) completed a Monte Carlo study in which they examined 10 unique measures that could be calculated using 2 × 2 contingency table data. In their simulation, Schraw et al. generated data for a 2 × 2 table simulating a 1,000-item test using 10,000 cases. The data were generated using a two-phase process. In brief, the responses distribution varied from case to case, but the aggregated data yielded 62.5% of responses in cell a and the remainder evenly distributed in cells b-d. This, according to Schraw et al., is the approximate distribution one would expect from a large sample of human participants. Although not all 10 measures represent independent psychological constructs, two important and distinct constructs were identified: sensitivity and specificity. *Sensitivity* represents the ability to accurately assess what is known. *Specificity* is the ability to accurately assess the unknown. Although Schraw et al.’s study was informative it was conducted using Monte Carlo techniques. Future research using human participant generated data to examine the validity of sensitivity and specificity as individual differences and psychological constructs is necessary.

### Conclusion

Knowledge monitoring is an important component of metacognition and self-regulated learning. As Bjork, Dunlosky, and Kornell (2013) stated, learning requires continual assessments and decisions about what has been studied and what should be studied next. The results of the current study suggest that the ability to discriminate what is known is an important part of the learning process and is perhaps a robust psychology construct worthy of further investigation in both psychological and education research.

## Footnotes

1 Italicized items in Appendix A represent content specific stimuli.

2 There are several hypotheses and theories as to the source of individuals’ feelings of knowing, judgments of learning, etc., but there are beyond the scope of this manuscript (however, cf. Dunlosky & Metcalfe, 2009).

## APPENDIX A

**Table:**

Knowledge monitoring assessment stimuli				
**1. accommodation** *suggestion connection relationship adjustment pronunciation*	**11. catharsis** *opposite rebellion approval representation release*	**21. entropy** *enclosure truth disorganization quality quantity*	**31. intrinsic** *real harmful internal implicit input*	**41. reprimand** *vision effect survey impetus criticism*
**2. algorithm** *trait anxiety rule contradiction survey*	**12. choleric** *pleasant abnormal steady diverse angry*	**22. exogenous** *expensive lively indecent generic external*	**32. locus** *location justice formula truth border*	**42. salience** *goal images importance insulation strength*
**3. angst** *anxiety consistency disease equilibrium anger*	**13. contiguous** *periodic sudden productive adjacent external*	**23. exemplar** *opposite rebellion release representation resolution*	**33. moratorium** *degree achievement reduction delay model*	**43. satiation** *fullness excellence degree responsive capability*
**4. antecedent** *dissimilar cause approval periodic norm*	**14. correlation** *imbalance generation relationship adjustment persuasion*	**24. generative** *similar sudden productive adjacent suggestive*	**34. myelination** *goal images importance insulation persuasion*	**44. seriation** *maintenance ordering scatter difference border*
**5. archetype** *linotype somatotype collotype teletype prototype*	**15. criterion** *kindness standard crossover critic endorsement*	**25. heterogeneous** *steady lively generic hopeless diverse*	**35. orthogonal** *dependable excessive stable dynamic perpendicular*	**45. sustaining** *adjusting continuing passive balance decreasing*
**6. assimilate** *appear impact absorb devote erode*	**16. diopter** *response estimation power gravity friction*	**26. hierarchy** *impairment vagueness value order apprehension*	**36. ossify** *focus harden classify organize inspire*	**46. synapse** *similarity slip space strength endorsement*
**7. attenuation** *reduction contradiction strength excellence generation*	**17. disequilibrium** *adjusting continuing formula imbalance norm*	**27. implacable** *average helpless indecent marginal relentless*	**37. peripheral** *marginal expand lively useful probable*	**47. tacit** *real harmful internal implied powerful*
**8. aversive** *valuable unpleasant average successful powerful*	**18. divergent** *dissimilar cause conditional periodic pleasant*	**28. incentive** *example reward imbalance rule potential*	**38. prototype** *example reward imbalance drive crossover*	**48. taxonomy** *anxiety categorization friction estimation truth*
**9. benevolence** *kindness learning obedience opportunity burden*	**19. efficacy** *capability inadequacy persistence character helplessness*	**29. incremental** *successful growing nearuseful hopeless*	**39. proximal** *valuable passive dependable emotional near*	**49. utility** *impairment vagueness model level value*
**10. capricious** *expensive cozy impulsive reasoned abnormal*	**20. elaborate** *adjust connect expand exclude endorse*	**30. intermittent** *reasoned critical external irregular passive*	**40. reactivity** *gravity usefulness justice resolution responsiveness*	**50. volition** *determination pronunciation modification integration burden*
